# West London Hospital

**Published:** 1902-04-26

**Authors:** 


### WEST LONDON HOSPITAL.

### ABOUT 300 GOVERNORS AT AN ANNUAL MEETING.

"Get a chairman with a name," said one of the speakers on Thursday, April 17th, "and you will draw a meeting." However this may be, the meeting was certainly drawn, and the large room in which it was held was packed so full that by 5 o'clock, the time advertised, it was thought there "might be standing room."

Besides the chairman's name, there was the attraction of the opening of a new ward at the top of the recently completed new wing. This was the "Annie Zunz ward," given by the trustees of the late Mr. Siegfried Rudolf Zunz; who have agreed to award £10,000, in annual instalments of £1,000, for the purpose. Two instalments have been already received. The ward, which was visited by a number of people both before and after the meeting, will accommodate 26 patients, four of whom will be children and the rest medical cases (women). It has recently been used for lectures in connection with the post-graduate college.

Mr. Leopold de Rothschild, J.P., D.L., took the chair, and with him on the platform were the Rev. Canon White, Mr. J. R. Gilbert, the secretary-superintendent, and others. Mr. Gilbert read the whole of the report, thereby taking the wind out of the chairman's sails, for, as he explained, he had prepared a speech based upon it, which was now useless. One point, he said, had struck him, namely, the increased cost for maintenance; this was due partly to the higher price of provisions, but chiefly to the greater expense of surgical instruments. Then he worked out a little sum bearing on the average stay of patients in the hospital, which is 20·7 instead of, as formerly, 22·9 days. As there were 2,243 patients, these two days, said Mr. Rothschild, represented the work of one man for eleven years, and this meant a great deal in the aggregate. He remembered the time when people went to hospital about as willingly as to prison. Now, however, there was more comfort and better advice there than in the richest man's house, where soft carpets and bed-hangings retarded recovery.

#### The Wants of the Hospital.

The wants of that hospital, the speaker went on, were manifold. These were a few: £63,000 to complete the buildings, and to provide the requisite number of beds and cots, viz, 250, to meet the needs of the sick and injured of the district. The more immediately pressing portion included the nurses' home, providing for each nurse the privacy of a room to herself; the laundry, post-mortem, and mortuary block, and the administrative block, completing the frontage to Hammersmith Road. £13,000 was required to pay off the debt, and £9,000 annually to maintain the present work of relieving about 2,000 in-patients and 30,000 out-patients, while £1,500 would be required to maintain the new ward. He would particularly point out the attraction of having one's name over a bed or a cot, and this could be secured for £10 10s., which would buy furniture for one bed. Seventy beds wanted endowment. He was more especially interested in hospitals from having had a son desperately ill; by God's providence he was now recovering. Later on, amid great applause, the Chairman declared the Annie Zunz ward open; he suggested that a letter should be sent to the trustees, saying that the visitors walked through the ward and were delighted with the structural and other arrangements, and were, further, much gratified that the trustees should have selected the West London Hospital for one of their awards. One of the trustees had intended being present, but he had been prevented, and he thought it would be gratifying to them to know how greatly their gift was appreciated by the inhabitants of Fulham and Hammersmith. He himself felt the greatest interest in the hospital, having known it from the first. He was not a "Hammersmith rough," but he often passed that way, and he always noticed that the working men looked up at the hospital with a kind smile; they showed their appreciation in their subscriptions as well.

Canon Fisher, towards the close of the meeting, which lasted an hour and a half, announced that the Chairman had given £100 as a donation to the hospital. Several speakers alluded to the great financial support accorded by the Rothschild family. The Right Hon. the Lord Rothschild was unanimously re-elected Hon. Treasurer of the hospital, and the whole of the retiring members of the board of management were also re-elected for the ensuing year. Refreshments preceded the meeting, which was followed by a programme of music.

